# A multihued sustainable appraisal of the electrochemical method for synchronized micro-estimation of the household drug Paracetamol with Aceclofenac or Dicyclomine

**DOI:** 10.1038/s41598-026-41215-w

**Published:** 2026-03-22

**Authors:** Aya R. Ahmed, Marwa A. A. Ragab, Mohamed A. Korany, Samar Abu Khashaba, Sara I. Aboras

**Affiliations:** Department of Pharmaceutical Analytical Chemistry, Faculty of Pharmacy, Alexandria University, Alexandria, Egypt

**Keywords:** Voltammetric method, Paracetamol, Aceclofenac, Dicyclomine, Quality and sustainability, Sustainable development goals, Chemistry, Health care

## Abstract

Paracetamol (PCT) is an analgesic and antipyretic that is consumed on a large scale and frequently formulated in fixed-dose combinations to enhance therapeutic efficacy. In this study, an Eco-benign differential pulse voltammetric method using an unmodified glassy carbon electrode (GCE) was developed for the simultaneous micro-estimation of PCT in two binary mixtures: with Aceclofenac (ACL) and with Dicyclomine (DIC). In the Britton–Robinson buffer (BRB) at pH 2, PCT and ACL exhibited well-resolved anodic peaks at 0.62 and 0.81 V, respectively, while at pH 9, PCT and DIC demonstrated oxidation peaks at 0.41 and 0.58 V, respectively. The validation of the proposed method was conducted in accordance with ICH guidelines and they are applied to pharmaceutical tablet formulations successfully. Linearity was achieved over concentration ranges of 0.2–25 µg·mL⁻¹ for PCT and ACL, and 1–25 µg·mL⁻¹ for PCT and DIC, with coefficients of correlation that exceed 0.9995. The method adheres to Green and White Analytical Chemistry principles by minimizing reagent consumption, analysis time and environmental impact. A multihued sustainability assessment was conducted using several greenness and sustainability metrics and compared with reported techniques. In addition, alignment with the United Nations Sustainable Development Goals was evaluated using the innovative Need, Quality and Sustainability (NQS) index. The results demonstrate that the proposed voltammetric approach is simple, rapid, cost-effective and suitable for routine quality control analysis of paracetamol-based binary mixtures.

**Supplementary Information:**

The online version contains supplementary material available at 10.1038/s41598-026-41215-w.

## Introduction

**Fig. 1:**
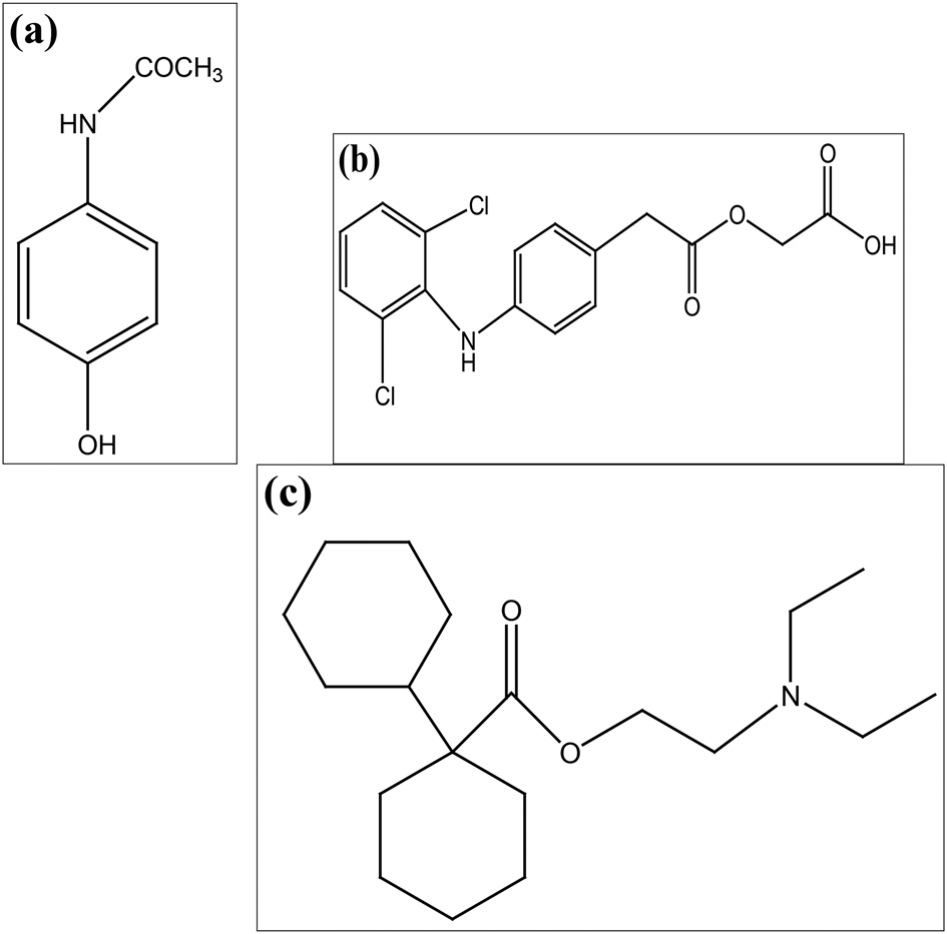
Chemical structures of (**a**) Paracetamol (PCT), (**b**) Aceclofenac (ACL) and (**c**) Dicyclomine (DIC).

Paracetamol (PCT), chemically known as N-(4-hydroxyphenyl) acetamide, Fig. 1a, is among the predominantly utilized analgesic and antipyretic agents across the globe. Its therapeutic action is primarily attributed to the inhibition of cyclo-oxygenase enzymes (COX-1, COX-2, and COX-3), leading to reduced prostaglandin synthesis and subsequent alleviation of pain and fever. Due to its efficacy and safety profile, PCT is commonly prescribed alone or in combination with other drugs for the treatment of mild to moderate pain, fever, and inflammatory conditions^1^.

Aceclofenac (ACL), a non-steroidal anti-inflammatory drug, is chemically described as 2-[2-[2-(2,6-dichloroanilino)phenyl]acetyl]oxyacetic acid, Fig. 1b. It exerts its analgesic and anti-inflammatory effects mainly through selective inhibition of the COX-2 enzyme, thereby reducing the formation of pro-inflammatory prostaglandins. ACL is widely used in the management of osteoarthritis, rheumatoid arthritis and other musculoskeletal disorders^2^.

Dicyclomine (DIC), chemically known as 2-(diethylamino)ethyl 1-cyclohexylcyclohexane-1-carboxylate, Fig. 1c, is an anti-cholinergic agent commonly prescribed for irritable bowel syndrome. It relieves gastrointestinal spasms by inhibiting muscarinic acetylcholine receptors, resulting in decreased intestinal motility and secretions^3^.

Combination drug therapy is increasingly employed to enhance therapeutic outcomes, target multiple pathological pathways and improve patient compliance^4^. In this context, PCT is frequently combined with ACL to provide synergistic analgesic and anti-inflammatory effects in conditions such as osteoarthritis and rheumatoid arthritis^5,6^. Additionally, PCT is co-administered with DIC for managing spasmodic pain associated with gastrointestinal, biliary and renal disorders as well as dysmenorrhea^7^.

A literature review has demonstrated that several methods of analysis have been put into action for determining the first combination therapy in a simultaneous manner, mixture 1, in different matrices, such as HPLC^8–12^, HPTLC^13,14^ and spectrophotometric methods^15–17^. In addition to that, a prior analysis of mixture 2 was carried out utilizing HPLC^18–20^, HPTLC^21^ and spectrophotometric^22,23^ methods.

Voltammetric techniques offer attractive alternatives due to their simplicity, sensitivity and low reagent consumption^24^. Numerous voltammetric methods have been reported for the determination of PCT^25–30^, ACL^31–33^ and DIC^34,35^ individually or in combination with other drugs. However, most of them rely on complex electrode modifications such as gold electrodes modified with self-assembled monolayers and gold nanoparticles, Fe(III)-encapsulated zeolite/graphite composite electrodes, boron-doped diamond electrodes or polymer/carbon nanotube–modified pencil graphite electrodes. While such modifications often enhance sensitivity, they increase method complexity, cost, preparation time and may suffer from limited reproducibility and surface fouling. In contrast, the present method employs a simple, unmodified glassy carbon electrode (GCE), while maintaining high sensitivity, offering a simpler, more reproducible, cost-effective, and practically robust alternative for routine analysis. Importantly, to the best of our knowledge, this work represents the first voltammetric method for the simultaneous determination of PCT in two binary mixtures, namely with ACL or DIC, without prior separation or electrode modification. This combination of simplicity, sensitivity and multicomponent capability highlights the novelty and practical significance of the proposed method.

In line with the concepts of Green Analytical Chemistry (GAC), an emerging trend in drug analysis is the development of multi-analyte wide-scope techniques. Herein, this study offers multi-analytes, which are preferred over methods using one analyte at a time (GAC principle 8). This leads to a reduction of waste (GAC principle 7). Besides, our method is devoid of derivatization and other extra steps in analysis (GAC principle 6), as they squander resources and reagents. In addition to conserving energy and time (GAC principle 9), the method uses aqueous buffer instead of toxic organic solvents (GAC principle 11), which results in less operator effort and solvent exposure (GAC principle 12)^36^. In addition to that, this study demonstrates how scientific innovation can be effectively combined with environmental sustainability and highlights the importance of alignment with the United Nations (UN) Sustainable Development Goals (SDGs)^37^.

As far as we know, this study offers the first voltammetric technique for the simultaneous micro-estimation of PCT in two binary mixtures with ACL or DIC. A significant advantages are offered by this proposed method, including rapid analysis within seconds, high specificity, operational simplicity and the utilization of a cost-efficient and environmentally friendly approach. These features make it highly suitable for routine application in quality control laboratories. Furthermore, the ability to perform a multicomponent determination of different formulations supports the maintenance of quality standards while the necessity for separate methods for every individual formulation is eliminated, ultimately streamlining analytical workflows and reducing labor, cost and analysis time. Besides, the suggested approach was thoroughly assessed and contrasted with reported techniques using the frameworks of GAC and White Analytical Chemistry (WAC), proving its dedication to efficiency and sustainability. The multihued sustainable appraisal is fulfilled using different tools, such as the Analytical Eco-scale^38^, Analytical GREEnnes (AGREE)^39^, ChlorTox^40^, RGB12^41^ and Blue Applicability Grade Index (BAGI)^42^. Additionally, alignment with the SDGs is assessed using the Need, Quality and Sustainability index (NQS)^43^.

## Experimental

### Instrument

Voltammetric measurements were carried out using a computer-controlled 797 VA Computrace analyzer (Metrohm, Herisau, Switzerland) fitted with a multimode electrode (MME). The three-electrode system consisted of an Ag/AgCl reference electrode saturated with 3 M potassium chloride (KCl), a platinum auxiliary electrode and a GCE as the working electrode. Sonication was performed using a J.P. SELECTA, S.A. ultrasonic bath (Abrera, Barcelona, Spain). Accurate weighing was achieved with a KERN & SOHN balance (GmbH, Balingen, Germany) and pH measurements were obtained using a pH meter manufactured by Crison Instruments, S.A., Barcelona.

### Material

Amriya Pharmaceutical Industries (Alexandria, Egypt) generously provided pure standards of PCT (99%), ACL (98%) and DIC (98.9%). The commercially manufactured combination dosage forms (Algic-P^®^ containing 500 mg PCT and 100 ACL and Cyclopam^®^ tablets containing 500 mg PCT and 20 mg DIC) weren’t available in the local market. Thus, laboratory-prepared tablets were formulated to represent these two mixtures in their formulated ratios. The laboratory-prepared tablets were assembled using individual commercially available products: Paracetamol^®^ tablets (500 mg PCT per tablet), Bristaflam^®^ tablets (100 mg ACL per tablet) and Spasmorest^®^ tablets (20 mg DIC per tablet). Paracetamol^®^ tablets (500 mg PCT) contain pregelatinized starch (maize), sodium metabisulfite, stearic acid and magnesium stearate.

Bristaflam^®^ tablets (100 mg ACL) contain microcrystalline cellulose, croscarmellose sodium, copovidone, talc, colloidal anhydrous silica, glycerol distearate, hypromellose, titanium dioxide and polyoxyl 40 (macrogol) stearate.

Spasmorest^®^ tablets (20 mg DIC) contain acacia, dibasic calcium phosphate, corn starch, lactose, magnesium stearate, pregelatinized corn starch and sucrose.

In addition to that, aluminum oxide was supplied by Cambrian Chemicals, while boric acid was purchased from El-Gomhoreya Pharmaceutical Chemicals Co., Egypt. Glacial acetic acid and HPLC-grade methanol were obtained from Gliwice (ul. Sowińskiego 11, Poland), whereas sodium hydroxide and ortho-phosphoric acid were sourced from El-Nasr Chemical Co., Alexandria, Egypt. Deionized water was consistently used throughout the experimental procedures to maintain accuracy and reproducibility.

### Stock and working solutions preparations

Stock solutions of PCT, ACL and DIC were prepared at a concentration of 1000 µg. mL^− 1^ using methanol as the solvent. These stock solutions were subsequently diluted with methanol to obtain working solutions at a concentration of 100 µg. mL^− 1^ for each drug. All stock and working solutions were stored at 4 °C under refrigerated conditions.

### Calibration curves construction

Calibration curves were prepared using the working solutions described earlier. For mixture 1, the analysis was performed in a Britton–Robinson buffer (BRB) at 0.04 M, containing acetic acid, ortho-phosphoric acid and boric acid, adjusted to pH 2. For mixture 2, the buffer pH was raised to 9 using 1 M sodium hydroxide to achieve the desired conditions. Aliquots from the working solutions of PCT, ACL and DIC were diluted with BRB to produce two sets of solutions, with concentration ranges of 0.2–25 µg. mL^− 1^ for mixture 1 and 1–25 µg. mL^− 1^ for mixture 2. These solutions were then used to construct the calibration curves.

### Polishing

The electrode surface was conditioned by polishing for one minute on a pad moistened with a dough of alumina–water. This step was followed by an additional one-minute polishing on a pad soaked with deionized water. Finally, the GCE was thoroughly rinsed with deionized water to remove any residual particles.

### Voltammetric method for differential pulse (DP)

**Fig. 2:**
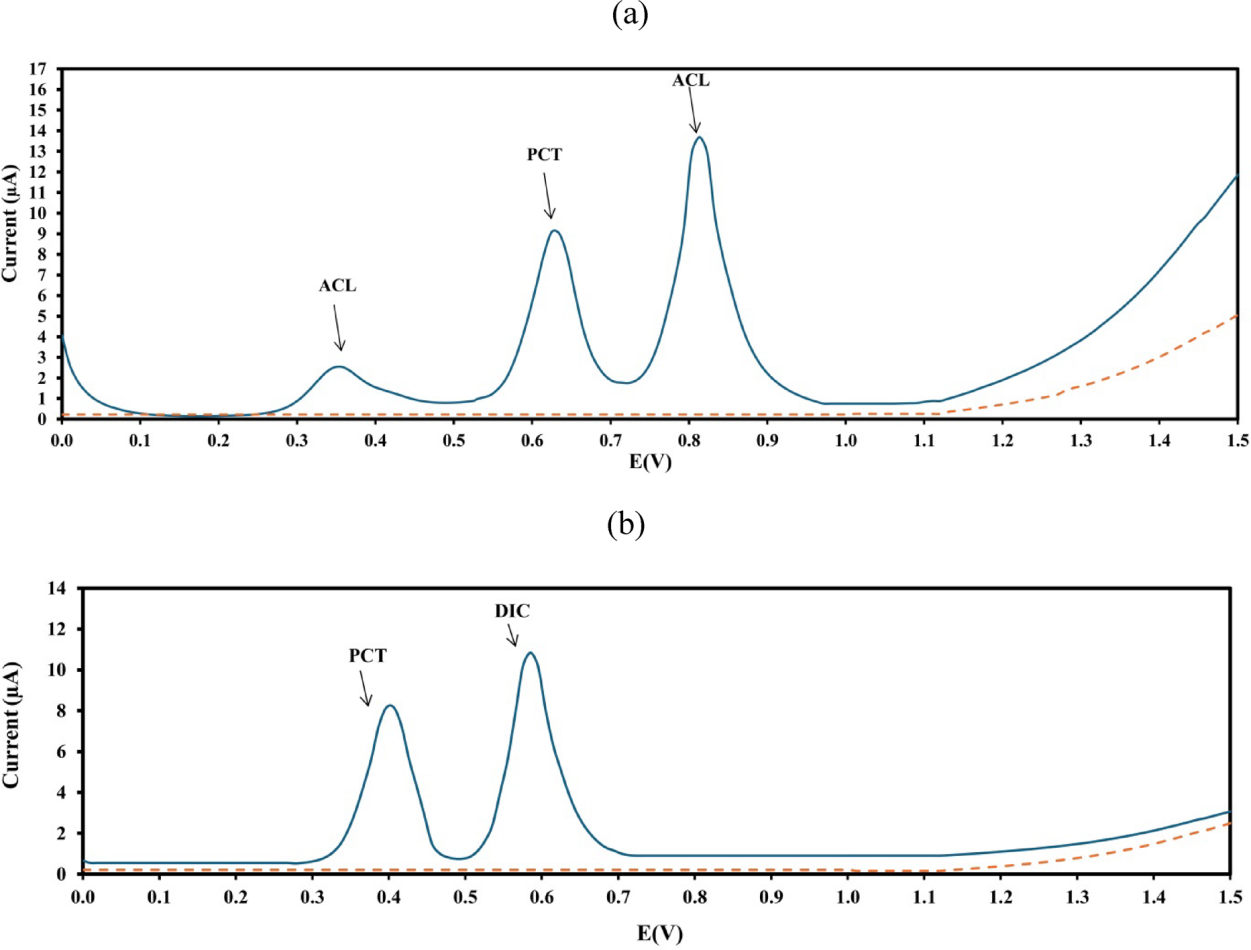
Differential pulse voltammograms of (**a**) mixture 1 (PCT-ACL) equivalent to 5:5 µg. mL^− 1^ using BRB at pH 2 (………), pulse amplitude of 0.05 V, pulse time of 0.03 s, voltage step of 9 mV, and voltage step time of 0.1 s. (**b**) mixture 2 (PCT-DIC) equivalent to 5:5 µg. mL^− 1^ using BRB at pH 9, pulse amplitude of 0.2 V, pulse time of 0.02 s, voltage step of 9 mV, and voltage step time of 0.2 s at a scan rate of 100 mVs^− 1^ versus Ag/AgCl reference electrode.

Following the transfer of the contents of each final flask to the electrochemical cell, differential pulse (DP) voltammograms were recorded using the working electrode. Pulse amplitudes of 0.05 V and 0.2 V were applied for mixture 1 and mixture 2, respectively. For both mixtures, voltammetric measurements were carried out over a potential range of 0 to + 1.5 V versus the Ag/AgCl reference electrode, at a scan rate of 100 mV s⁻¹. For mixture 1, PCT exhibits an anodic peak at 0.62 V and ACL showed two anodic peaks at 0.36 V and 0.81 V when measured using BRB at pH 2. The major peak for ACL at 0.81 V was used for its quantification. For mixture 2, PCT appears at 0.41 V and the DIC anodic peak is at 0.58 V at pH 9, as shown in Fig. 2. The voltammograms were blank corrected.

### Application of the proposed method

For mixture 1, five commercial tablets of Paracetamol^®^ and Bristaflam^®^ were accurately weighed and finely powdered together. A quantity of the powder equivalent to 500 mg of PCT and 100 mg of ACL was transferred into a 100 mL volumetric flask. The contents were mixed with a proper quantity of methanol, then sonicated for 20 min to ensure complete dissolution. The solution was brought up to volume with methanol, then filtered to construct a solution equivalent to 5000 and 1000 µg. mL^− 1^ for PCT and ACL, respectively. After appropriate dilutions, the final concentrations achieved were 5 µg. mL^− 1^ of PCT and 1 µg. mL^− 1^ of ACL using BRB at pH 2.

For mixture 2, five commercial tablets of Paracetamol^®^ and Spasmorest^®^ were accurately weighed and finely powdered together. An amount corresponding to 500 mg of PCT and 20 mg of DIC was combined in a 100 mL volumetric flask with a proper quantity of methanol. After 20 min of sonication, the volume was completed with methanol and then filtered. The resulting solution had concentrations of 5000 µg. mL^− 1^ of PCT and 200 µg. mL^− 1^ of DIC. Further dilutions were performed, yielding final concentrations of 25 µg. mL^− 1^ of PCT and 1 µg. mL^− 1^ of DIC using BRB at pH 9.

## Results and discussion

### Method development and optimization

Various factors influencing each drug’s peak current (I_p_) and potential (E_p_) were examined. BRB is a universal supporting electrolyte in electroanalytical techniques because it provides a wide stable pH control across a broad range of pH (2–12), unlike conventional buffer systems that are limited to either acidic or alkaline regions. This wide buffering capacity allows systematic investigation of pH-dependent electrochemical behavior using a single buffer composition, thereby simplifying experimental design and improving method generalizability. Moreover, it provides a stable electrolyte environment without introducing interfering redox reactions for DP voltammetric determination of the cited drugs.

#### Effect of buffer pH

**Fig. 3:**
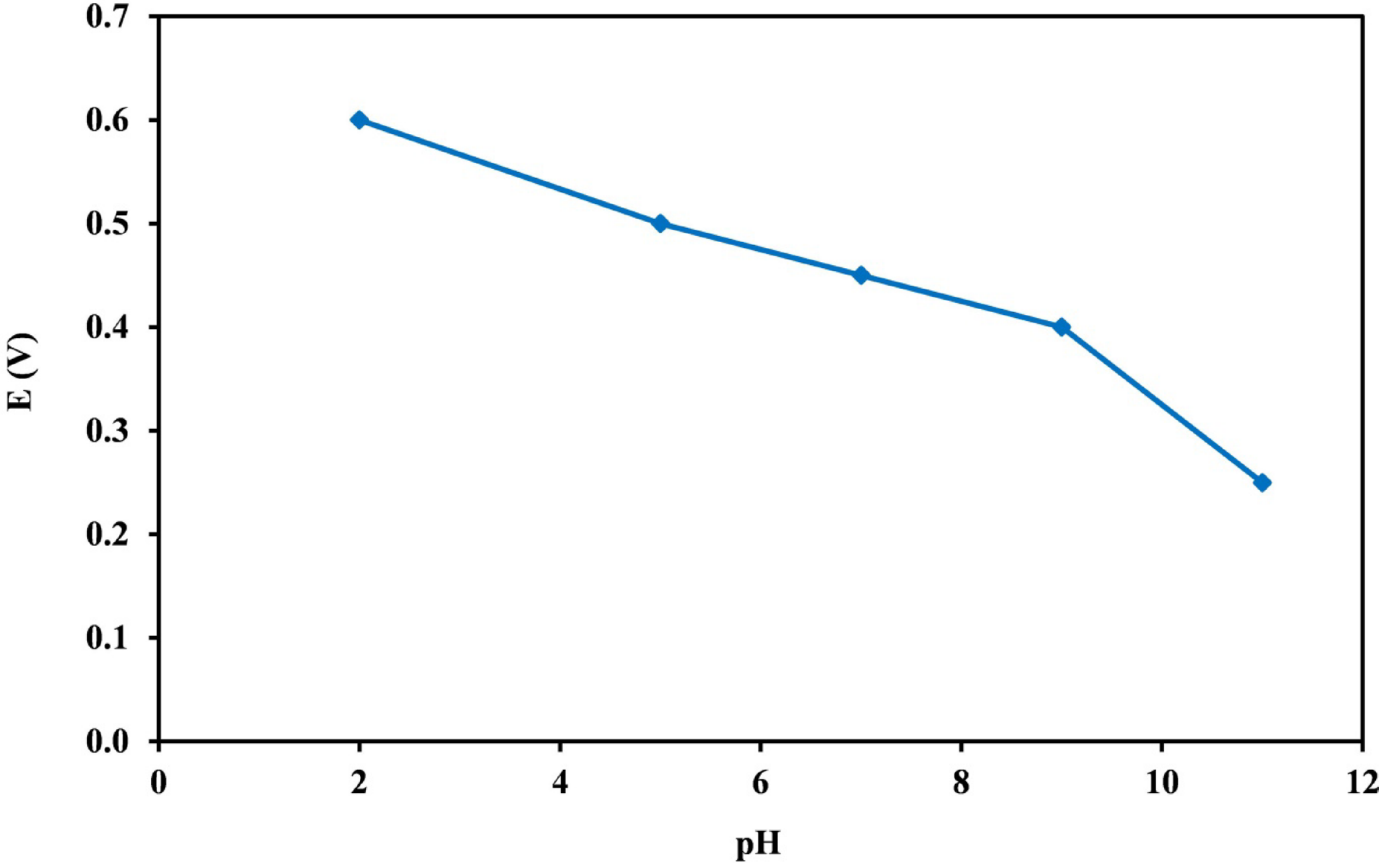
Shifting of the potential of the PCT anodic peak with changing pH of BRB at a scan rate of 100 mVs^−1^ versus Ag/AgCl reference electrode.

The influence of the buffer solution’s pH on the Ep was examined using BRB in the range of pH (2–11). The oxidative Ep varied throughout the entire pH range. As shown in Fig. 3, the electrochemical oxidation of PCT is clearly pH-dependent, with the anodic Ep shifting toward more negative values as the pH increases, indicating the involvement of protons in the redox process^28,29^. At higher pH, deprotonation of the phenolic group facilitates electron transfer, lowering the energy required for oxidation. The Ep shows a linear relationship with pH, with a slope close to the theoretical Nernstian value of − 59 mV per pH unit, suggesting that the oxidation involves an equal number of electrons and protons, likely two of each, leading to the formation of N-acetyl-p-benzoquinone imine. These observations are consistent with previous reports^26,29,30^ and confirm the proton-coupled electron transfer mechanism of PCT oxidation.

In contrast to PCT, ACL did not show well-defined oxidation peaks at pH values above 7 when measured at the GCE. This lack of a voltammetric response can be attributed to pH-dependent changes in molecular speciation and interfacial electron-transfer kinetics. ACL contains a carboxylic acid group (pK_a_ ≈ 4.7–5.0), which is predominantly deprotonated at pH values above 7. The resulting anionic species have reduced adsorption affinity for the hydrophobic glassy carbon surface and are subject to electrostatic repulsion from negatively charged surface oxides, leading to lower interfacial concentration and hindered electron transfer. Furthermore, under alkaline conditions, limited proton availability makes proton-coupled oxidation pathways unfavorable, causing the oxidation potential to shift beyond the accessible potential window. Adsorption of oxidation products may also contribute to surface passivation, further suppressing measurable peaks. Together, these factors explain the disappearance of voltammetric signals for ACL at pH > 7^31,44^, while the DIC peak disappeared at a pH less than 7 upon their oxidation on GCE, because the requirement of alkaline pH for the voltammetric oxidation of DIC can be attributed to its basic tertiary amine functionality. At low and neutral pH values, the amine group is predominantly protonated, which suppresses electron transfer by reducing the availability of the nitrogen lone pair. Under alkaline conditions, deprotonation of the amine occurs, enabling oxidation at the nitrogen center and enhancing adsorption and electron-transfer kinetics at the GCE. Consequently, well-defined oxidation peaks are observed only in basic media, which coincides with previous reports of voltammetric analysis of this drug on modified GCE^34,35^. Therefore, mixture 1 was analyzed in acidic conditions while mixture 2 was detected in basic one as shown in Figs. S1a and S2a.

In Fig. S1a, PCT has a maximum I_p_ response at pH 2, while the I_p_ response above this pH value showed lower values. ACL also provided the greatest response at pH 2. PCT exhibits an anodic peak at 0.62 V when measured using BRB at pH 2, while ACL has an anodic peak at 0.81 V at pH 2. Based on this, the choice fell on pH 2 for the analysis of mixture 1.

As seen in Fig. S2a, it was discovered that the electrocatalytic oxidation of DIC at the GCE depended on the aqueous solution’s pH value and that the solution’s pH had a notable impact on the I_P_ of DIC oxidation. The highest I_p_ of DIC was noted at pH 9. To achieve the highest level of bio-activity and ideal sensitivity, pH 9 was the optimum pH value for the analysis of mixture 2 where the PCT anodic peak appeared at 0.41 V, while that for DIC was obtained at 0.58 V.

#### Effect of pulse amplitude

Pulse amplitude was tested over a potential range of (0.01–0.3) V and optimal responses were observed at 0.05 V and 0.2 V for the simultaneous analysis of the drugs in mixture 1 and mixture 2, respectively, as illustrated in Figs. S1b and S2b.

#### Effect of pulse time

Pulse time was measured (0.01–0.05) seconds. For the two studied mixtures, the ideal pulse times were determined to be 0.03 s and 0.02 s for mixture 1 and mixture 2, respectively. The I_p_ responsiveness decreased with pulse times other than these values, as found in Figs. S1c and S2c, respectively, meaning that the chosen pulse times for both mixtures were the optimum for our analysis.

#### Effect of voltage step

The effect of the voltage step was investigated over a range of (1–9) mV. The highest I_p_ for both mixtures was obtained at a voltage step of 9 mV, as illustrated in Figs. S1d and S2d.

#### Effect of voltage step time

The voltage step time was evaluated within the range of (0.1–0.4) seconds. It was found that the use of 0.1 s for mixture 1, while 0.2 s for mixture 2 was the optimum voltage step time as these values resulted in the highest I_p_ for both mixtures, as shown in Figs. S1e and S2e, respectively.

Finally, the optimum conditions for mixture 1 and mixture 2 are summarized in Table 1.

**Table 1:** Optimum conditions selected for analysis of mixture 1 and mixture 2.

Parameter	Mixture 1	Mixture 2
Buffer pH	2	9
Pulse amplitude (V)	0.05	0.2
Pulse time (s.)	0.03	0.02
Voltage step (mV)	9	9
Voltage step time (s.)	0.1	0.2

### Cyclic voltammetric study

**Fig. 4:**
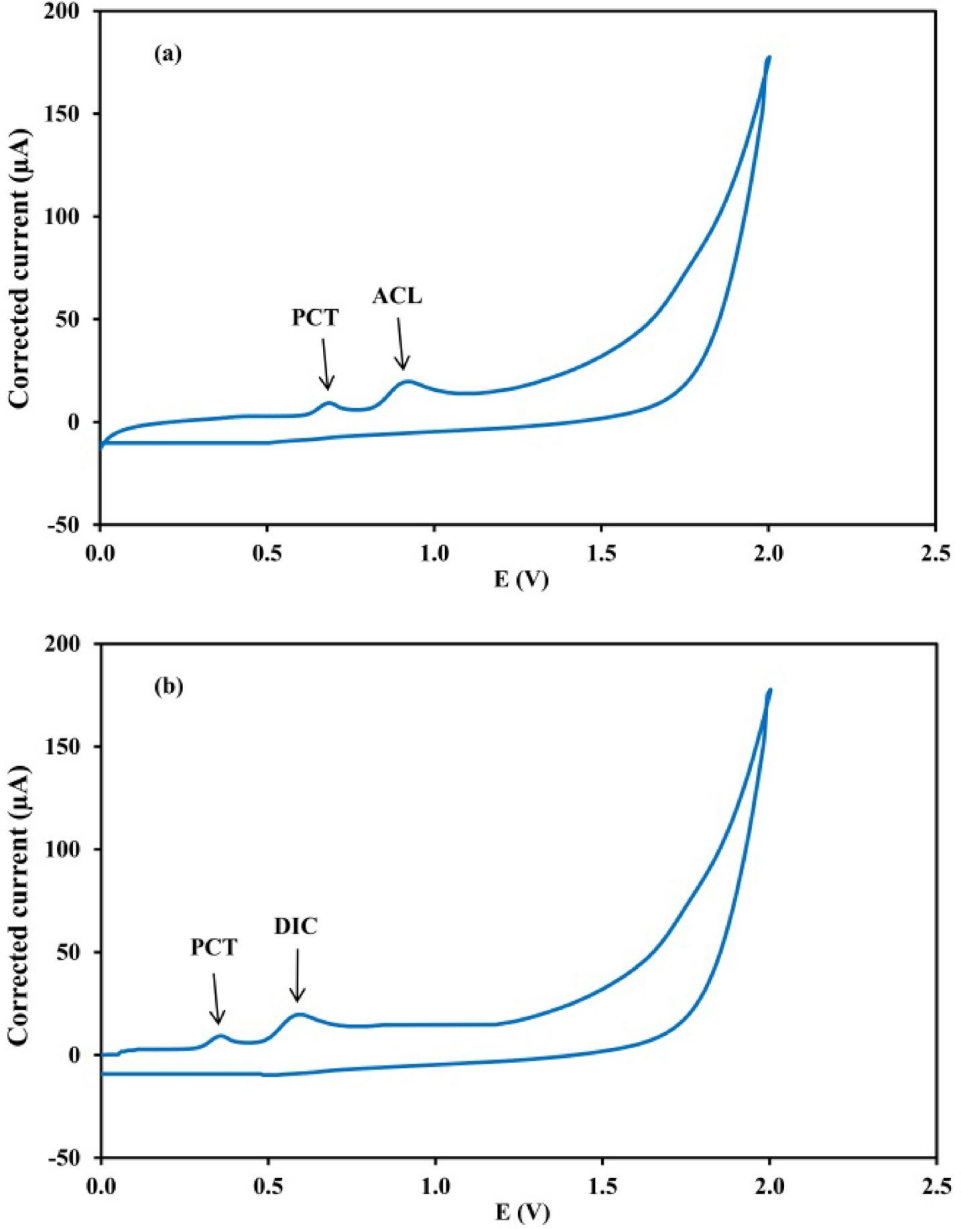
Cyclic voltammograms of (**a**) mixture 1 using BRB at pH 2, pulse amplitude of 0.05 V, pulse time of 0.03 s, voltage step of 9 mV and voltage step time of 0.1 s and (**b**) mixture 2 using BRB at pH 9, pulse amplitude of 0.2 V, pulse time of 0.02 s, voltage step of 9 mV and voltage step time of 0.2 s, equivalent to 10 µg. mL^− 1^ for each drug at a scan rate of 100 mVs^− 1^ versus Ag/AgCl reference electrode.

Cyclic voltammetry was employed as an analytical tool to investigate the electrode processes. The resulting cyclic voltammograms for the two pharmaceutical mixtures are presented in Fig. 4. The cyclic voltammetric oxidation of PCT, ACL and DIC at GCE exhibits irreversible behavior, as evidenced by the presence of a single anodic peak for each drug and the absence of a corresponding cathodic peak in the reverse scan.

For PCT, the oxidation involves a two-electron, two-proton transfer, forming the highly reactive intermediate N-acetyl-p-benzoquinone imine, which rapidly undergoes hydrolysis in acidic media or hydroxylation in alkaline media^45^. Previous studies on PCT oxidation have shown that the anodic peak potential shifts to less positive values with increasing scan rate, which is characteristic of an irreversible electron-transfer process. Additionally, the anodic peak current exhibited a linear relationship with the square root of the scan rate, further confirming the irreversible nature of the electrochemical oxidation of PCT and that the oxidation process is predominantly diffusion-controlled^25,28,46^.

The electrocatalytic oxidation of ACL at the GCE proceeds via an irreversible electrode process, as evidenced by the appearance of a single anodic peak and the absence of a corresponding cathodic peak in the reverse scan, in agreement with a previous report^47^. This behavior can be attributed to an initial anodic oxidation step that generates a chemically unstable aminophenol intermediate, which undergoes further oxidation to form a quinone-imine derivative, thereby preventing the reverse electrochemical reaction^31–33^.

According to a previous report, the electrochemical oxidation of DIC proceeds via an irreversible electrode process. The progressive increase in peak current at higher scan rates, together with the scan-rate dependence of the response, indicates that the reaction is predominantly governed by diffusion-controlled mass transport. In addition, DIC oxidation occurs predominantly at the tertiary amine group to form stable non-electroactive products, preventing the appearance of cathodic peaks and confirming the irreversible nature of their electrochemical oxidation^35^.

### Electrode reaction

The electrochemical oxidation of PCT is clearly pH-dependent, with the anodic Ep shifting toward more negative values as the pH increases, indicating the involvement of protons in the redox process^28^. PCT, a phenolic compound, undergoes electrochemical oxidation at the aromatic hydroxyl group, involving a two-electron, two-proton process that yields N-acetyl-p-benzoquinone imine. This reaction involves the irreversible transfer of two electrons, accompanied by the simultaneous release of two protons, as previously described in the voltammetric analysis of PCT on the GCE, Fig. S3a^25^.

Previous electrochemical studies have shown that ACL undergoes a multistep oxidation process at carbon electrodes, involving proton-coupled electron transfer. ACL oxidation includes an initial anodic oxidation step that generates aminophenol intermediate through hydroxylation, which undergoes further oxidation to form a quinone-imine derivative, Fig. S3b^31–33^. This multistep behavior gives rise to two anodic peaks: a minor peak at 0.36 V and a major peak at 0.81 V. The major peak was selected for quantitative analysis because it exhibits a higher I_P_ response and a linear increase with drug concentration, whereas the minor peak shows limited analytical significance.

Also, DIC undergoes irreversible oxidation primarily via the tertiary amine group which is surrounded by two electron-donating groups (2 ethyl groups), thus increasing the basicity and facilitating the loss of electrons and the oxidation reaction through a proton-coupled electron transfer process. The oxidation generates a radical cation that may undergo further chemical reactions or adsorb to the electrode surface, as indicated from previous reports, Fig. S3c^34,35^.

### Method validation

The proposed method was granted validation with respect to the guidelines of ICH^48^.

#### Linearity

**Fig. 5:**
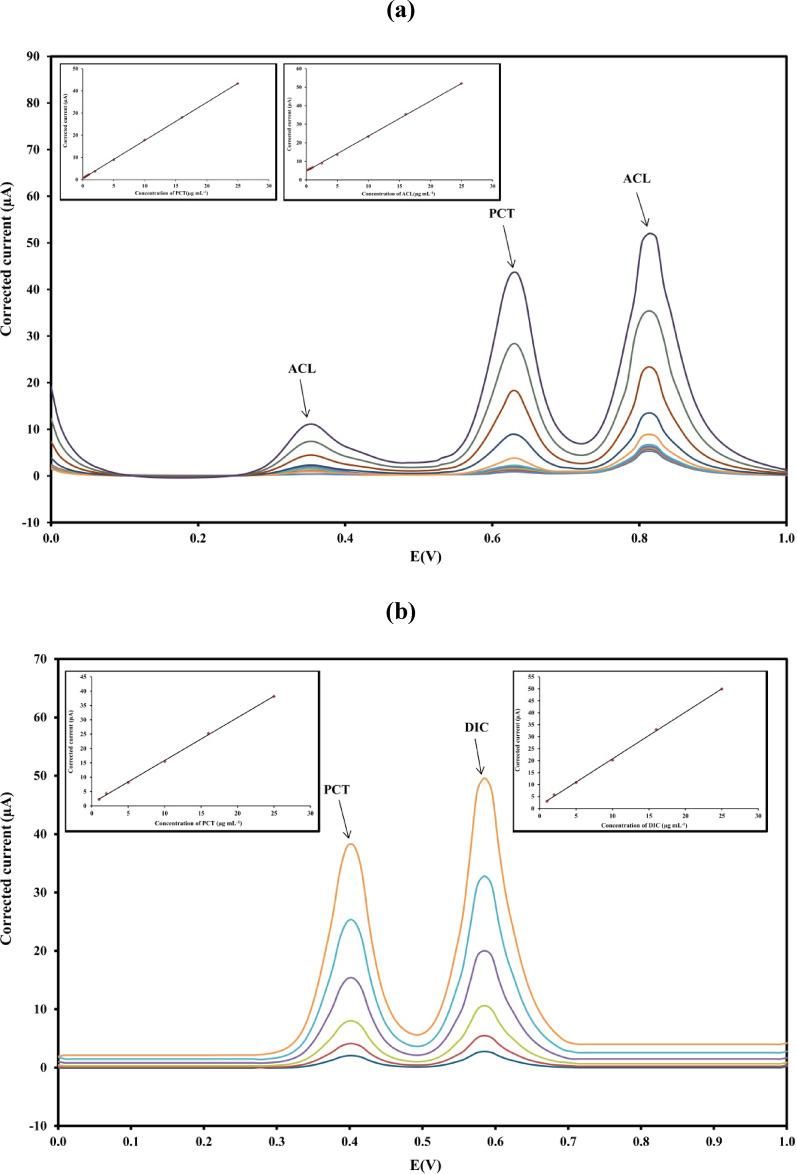
Differential pulse voltammograms corresponding to different concentrations of (a) mixture 1 [0.2–25 µg. mL^− 1^] using BRB at pH 2, pulse amplitude of 0.05 V, pulse time of 0.03 s, voltage step of 9 mV and voltage step time of 0.1 s and (b) mixture 2 [1–25 µg. mL^− 1^] using BRB at pH 9, pulse amplitude of 0.2 V, pulse time of 0.02 s, voltage step of 9 mV and voltage step time of 0.2 s at a scan rate of 100 mVs^−1^ versus Ag/AgCl reference electrode. The insert represents the corresponding calibration curve.

Under the optimized experimental conditions, a clear linear correlation was observed between the I_p_ and the concentration of each drug, as illustrated in the DP voltammograms in Fig. 5. The correlation coefficient (r^2^) exceeded 0.9995, indicating excellent linearity for the analyzed drugs. Detailed linearity data, along with associated statistical parameters for the method, are provided in Table 2.

**Table 2:** Regression and statistical parameters for the determination of mixture 1 and mixture 2 utilizing the proposed voltammetric method.

Parameter	Mixture 1		Mixture 2	
	PCT	ACL	PCT	DIC
*Linearity range	0.2–25	0.2–25	1–25	1–25
a ^a^	0.52 × 10^− 6^	4.67 × 10^− 6^	0.83 × 10^− 6^	1.10 × 10^− 6^
Sa ^b^	0.03	0.04	0.16	0.19
b ^c^	1.71	1.97	1.62	1.96
Sb ^d^	0.01	0.01	0.02	0.02
S_y/x_^e^	0.14	0.35	0.46	0.37
r ^f^	0.9999	0.9998	0.9995	0.9997
LOD ^g^	0.06	0.07	0.33	0.32
LOQ ^h^	0.18	0.20	0.99	0.97

*Linearity range (µg. mL^− 1^).

^a^ Intercept. ^b^ Standard deviation of the intercept.

^c^ Slope. ^d^ Standard deviation of the slope.

^e^ Standard deviation of residuals. ^f^ Correlation coefficient.

^g^ LOD = Limit of detection (µg. mL^− 1^). ^h^ LOQ = Limit of quantitation (µg. mL^− 1^).

#### Limit of detection and limit of quantitation

The standard deviation of the intercept (S_a_), together with the slope, was used to calculate the limits of detection (LOD) and quantification (LOQ) according to the following formulas:

LOD = 3.3 × σ / S

LOQ = 10 × σ / S

Where σ is the standard deviation of the intercept, S is the slope.

Table 2 presents the calculated minimum detectable amounts based on the described parameters. The method demonstrates sufficient sensitivity, as reflected by the low LOD and LOQ values.

#### Accuracy and precision

To evaluate accuracy as well as intra-day and inter-day precision, three different concentrations of each of the two investigated mixtures were analyzed. The analytical outcomes of these experiments are summarized in Table 3. The low percentage error (%Er) and the relative standard deviation (%RSD) values below 2% demonstrate the high accuracy and precision of the proposed method for the determination of both mixtures.

**Table 3:** Evaluation of accuracy and precision for the determination of mixture 1 and mixture 2 using the proposed voltammetric method.

	Concentration (µg. mL^− 1^)		Accuracy					Precision						
			Mean % recovery ^a^		E_*r*_% ^b^			Intra-day RSD%^c^			Inter-day RSD%^c^			
Mixture 1	PCT	ACL	PCT	ACL	PCT	ACL		PCT	ACL		PCT	ACL		
	1	20	99.27 *±* 0.43	99.81 *±* 0.39	-0.73	-0.19		0.58	0.62		0.75	0.41		
	5	1	100.44 *±* 0.58	99.39 *±* 0.68	0.44	-0.61		0.75	0.58		1.05	0.70		
	20	1	99.34 *±* 0.58	99.67 *±* 0.45	-0.66	-0.33		0.68	0.72		0.79	0.69		
Mixture 2	PCT	DIC	PCT	DIC	PCT	DIC		PCT	DIC		PCT	DIC		
	1	16	101.03 *±* 0.27	98.58 *±* 0.41	1.03	-1.42		0.93	0.84		0.83	0.66		
	10	10	99.63 *±* 0.59	99.79 *±* 0.15	-0.37	-0.21		0.52	0.92		0.39	0.93		
	16	1	100.27 *±* 0.50	99.34 *±* 0.94	0.27	-0.66		0.22	0.79		0.36	0.70		

*The ratio of the synthetic mixtures is 1:20, 5:1, 20:1 for mixture 1 and 1:16, 10:10, 16:1 for mixture 2.

^a^ Mean recovery of the found concentrations± standard deviation for three determinations (*n* = 3).

^b^ % Relative error.

^c^ % Relative standard deviation.

#### Specificity

The method’s specificity is demonstrated by the clear resolution and non-overlapping anodic peaks of each drug within the binary mixtures with well-separated oxidation potentials and no mutual interference. Additionally, common tablet excipients did not produce any detectable voltammetric signals at the potential ranges of interest, confirming that the proposed method is specific for the simultaneous determination of the studied drugs in pharmaceutical formulations.

#### Robustness

No significant changes in I_p_ or E_p_ were observed when the buffer pH was deliberately varied by ± 0.2, as shown in Table S1 of the supplementary material. The stability of these responses indicates that the method is robust, confirming that the optimized procedure is reliable for the analysis of each mixture.

#### Stability

The stock and working solutions of each drug in methanol remained stable for at least one week when stored in the refrigerator at 4 °C. Additionally, the stability of the final measured standard solutions in BRB was assessed. No significant change in I_p_ or E_p_ was observed over ~ 2 h at pH 2 and pH 9 when compared to the freshly prepared solution, indicating that the drugs are stable under the applied experimental conditions.

### Assay of the proposed method on the laboratory-prepared tablets

The tablet solutions prepared according to Sect. (Application of the proposed method) were analyzed to assess the applicability of the proposed method. The results showed high accuracy, with %Er below 2%, and excellent precision as indicated by a %RSD of less than 2%. Moreover, no interference from co-formulated excipients in the tablets was detected, allowing for the direct quantification of all targeted drugs and demonstrating the method’s specificity as illustrated in Fig. S4 and summarized in Table 4. Moreover, our method is compared with other reported methods; it was found that t-test and F-test values for mixture 1 and mixture 2 were lower than the tabulated values at p *≤* 0.05, indicating that there was no significant difference between the proposed method and the other reported methods, as shown in Table S2.

**Table 4:** Assay results on laboratory-prepared tablets using the proposed voltammetric method.

	*Concentrations taken (µg. mL^− 1^)		^a^ Mean% Recovery ± SD		^b^ % RSD		^c^ %Er	
Mixture 1	PCT	ACL	PCT	ACL	PCT	ACL	PCT	ACL
	5	1	99.92 ± 0.28	99.67 ± 0.19	0.28	0.19	-0.08	-0.33
Mixture 2	PCT	DIC	PCT	DIC	PCT	DIC	PCT	DIC
	25	1	99.88 ± 0.85	100.02 ± 0.22	0.85	0.22	-0.12	0.02

*The ratio of the synthetic mixtures is 5:1 for mixture 1 and 25:1 for mixture 2.

^a^ Mean recovery of the found concentrations ± standard deviation for six determinations (*n* = 6).

^b^ % Relative standard deviation.

^c^ % Relative error.

### Comparison with reported methods

To emphasize the distinctiveness of the proposed method, a comparative evaluation with previously reported electrochemical and spectrophotometric methods is presented in Table S3. Most existing electrochemical approaches rely on sophisticated electrode modifications which, despite enhancing sensitivity, increase method complexity, cost, preparation time and may suffer from limited reproducibility and electrode fouling^29,33^. In contrast, the proposed method utilizes a bare GCE, offering a simpler, cost-effective and more reproducible strategy while maintaining high analytical performance with comparable sensitivity and adhering to GAC principles as it eliminates chemical modification steps and additional reagents.

Furthermore, although spectrophotometric methods are widely used, they often show lower sensitivity and require laborious mathematical treatments to resolve overlapping signals, particularly at trace levels^23,49^. Voltammetric techniques, which directly probe electron-transfer processes, offer superior sensitivity and inherent selectivity through characteristic peak potentials with minimal sample preparation and reliable performance in colored or complex matrices.

### Multihued sustainable assessment (green, blue, and white assessment)

In recent years, Eco-friendly and sustainable strategies have received considerable attention across a range of analytical techniques, including spectroscopy^50^, spectrofluorimetric^51^, HPLC^52–56^, HPTLC^57^ and capillary electrophoresis^58,59^. Analytical methods must comply with environmental safety standards and support the achievement of SDGs. In this context, the proposed method’s validation criteria, ecological impact (greenness), practical feasibility (blueness) and sustainability (whiteness) were extensively brought into comparison with previously reported HPLC (PCT-ACL) and (PCT-DIC), respectively^11,19^ and HPTLC (PCT-ACL)^13^ methods for the mixtures 1 and 2, Table 5. Various assessment tools were used to evaluate these characteristics, including the Eco-scale metric^38^, AGREE^39^ and ChlorTox^40^. In addition, the BAGI was employed to assess the method from both practical and economic viewpoints^42^. The RGB model was further applied to provide an integrated evaluation encompassing all relevant dimensions of these assessment paradigms. Together, these tools enabled a thorough appraisal of the method’s environmental impact and sustainability across different analytical stages, allowing its greenness and sustainability profile to be readily understood. Adherence to Eco-friendly principles in analytical method development prior to laboratory implementation is essential to reduce the environmental release of hazardous substances. Furthermore, the integration of multiple assessment frameworks to evaluate both the greenness and blueness of analytical methods is highly recommended as a standard component of method validation procedures^41^.

**Table 5:** Comparison of the proposed voltammetric method for the determination of PCT with ACL or DIC with other reported chromatographic methods.

Point of comparison	Proposed voltammetric method						HPLC (PCL-ACL)^11^		HPTLC (PCL-ACL)^13^		HPLC (PCL-DIC)^19^	
	PCT	ACL	PCT		DIC		PCL	ACL	PCL	ACL	PCL	DIC
Linearity µg. mL^− 1^	0.2–25		1–25				9–21	1.8–4.2	20–150	5-100	10–50	100–500
LOD µg. mL^− 1^	0.06	0.07	0.33		0.32		0.03	0.05	0.697	0.231	*N/M	
LOQ µg. mL^− 1^	0.18	0.2	0.99		0.97		0.1	0.16	0.425	0.114	N/M	

Applications	Tablets						Tablets		Tablets		Tablets	
Analytical eco-scale score	87						82		78		76	
AGREE												
ChlorTox scores (WHN Model)	0.04 < 0.1 gram neglected						3.42		0.36		5.26	
BAGI												
RGB and WAC score	95.9%						92.9%		93.0%		92.5%	

*N/M: Not mentioned.

The analytical Eco-scale relies on the imposition of penalty points on analytical process parameters that deviate from the optimal green analysis. Different parameters and analytical processes are compared using this method. The analytical Eco-scale is regarded as a useful, semi-quantitative tool, as described in Table S4. Our method has deservedly achieved the highest penalty points (87 points), due to its lower energy consumption, greener solvents and simpler instrumentation.

The AGREE calculator provides an automated and user-friendly approach for assessing method greenness and identifying areas for improvement. Compared with the Analytical Eco-scale, AGREE enables easier generation of comparable greenness scores with minimal effort^56^ and presents the results as a clock-like diagram that intuitively reflects the twelve principles of GAC Table 5. The proposed method achieved a higher AGREE score than reported methods, primarily due to the use of greener solvents, lower energy consumption and higher analytical throughput.

The ChlorTox scale is a widely accepted standard for assessing the greenness of analytical methods by quantifying the relative hazards of different reagents. The ChlorTox value is calculated using the following equation:

$$ChlorTox{\text{ }} = \frac{{CH_{{sub}} }}{{CH_{{CHCl3}} }}.m_{{sub}}$$

Here, CH_sub_ denotes the overall chemical hazard of the substance being evaluated, CH_CHCl₃_ represents the chemical hazard of chloroform used as a reference standard, and m_sub_ corresponds to the mass of the material under assessment.

The safety data provided by Sigma-Aldrich were used to calculate the CH_sub_ and CH_CHCl₃_ values according to the Weighted Hazards Number (WHN) methodology, which assesses overall chemical risk by integrating multiple safety-related parameters^40^. Precise estimation of reagent consumption at each step of the analytical procedure including method calibration, instrument preparation and rinsing, is essential to avoid underestimating the total procedural hazard. As shown in Table S5, the ChlorTox evaluation revealed that the reported chromatographic methods had the highest cumulative ChlorTox scores, mainly attributable to their extensive use of organic solvents. Conversely, the proposed voltammetric approach relies on an aqueous buffer system, rendering it more environmentally benign. These findings underscore the method’s advantage in terms of sustainability and operational efficiency.

For evaluating blueness, the innovative BAGI software offers a simple approach to assessing an analytical method’s efficiency. It helps identify both the advantages and limitations of a technique in terms of practical use and feasibility. Using this tool, the proposed method attained a BAGI score of 85, reflecting the highest level of practicality and applicability compared with other techniques, largely due to its high hourly throughput, as summarized in Table 5.

Given the limitations of individual sustainability assessment tools, the RGB multi-criteria model was applied to provide a balanced evaluation of analytical performance, GAC principles and economic aspects. As presented in Table 6 and Fig. S5, the proposed method achieved the highest blueness (96.5), greenness (95.0), and redness (96.3) scores, resulting in the highest overall whiteness score (95.9) compared with previously reported methods^11,13,19^. This superior performance is mainly attributed to the markedly shorter analysis time of the proposed voltammetric method (2 min) relative to chromatographic methods (8–14 min), which significantly reduced reagent consumption, waste generation and energy requirements while maintaining excellent accuracy, precision and sensitivity.

**Table 6:**
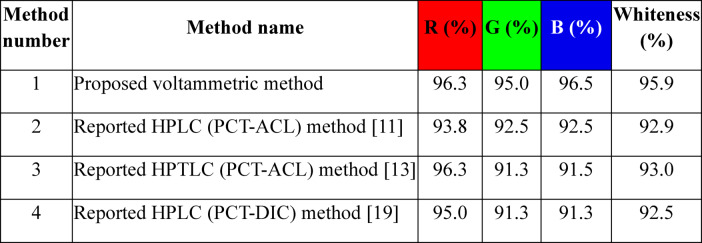
RGB profiles of the proposed voltammetric method compared with other reported chromatographic methods^11,13,19^. *Whiteness % indicates the arithmetic mean of the three other % (red, green and blue).

Ultimately, through a comprehensive, multi-criteria approach that integrates key factors such as validation requirements, cost effectiveness and practical applicability, the proposed voltammetric method demonstrated clear advantages over alternative techniques for the analysis of binary mixtures in laboratory-prepared tablets. The method achieved the highest scores for both whiteness and practical performance, indicating that it represents a more efficient and environmentally sustainable analytical option.

### Integration of sustainability objectives

Sustainability in analytical methodologies encompasses more than ecological considerations; these methods must be intentionally developed to contribute to and promote the achievement of SDGs^37,60^. The value of environmentally and socially conscious chemical practices has been acknowledged for decades, influencing processes at all levels from experimental laboratory work to large-scale industrial production^61,62^. Although various tools and strategies have been developed to encourage safer and more sustainable chemical practices, they often lack a direct link to the UN SDGs. This initiative seeks to close that gap by directly connecting awareness and innovation in green chemistry and sustainability to the SDGs^37,60^.

The electrochemical approach for multidrug analysis presented in this work combines scientific advancements with international sustainability initiatives to provide strong support for the SDGs. This is exemplified in Table S6. This study made a substantial contribution to sustainability in the scientific community by meeting 13 of the 17 SDGs and achieving a sustainability score of 76.0%.

Moreover, the NQS index is a state-of-the-art assessment tool developed to evaluate the evolving concept of “Analytical Chemistry for Sustainability.” This is accomplished by combining three core components: ‘Need’ (based on Koel’s pyramids), ‘Quality’ (evaluated using the WAC concept) and ‘Sustainability’ (aligned with the 17 SDGs), resulting in a unified percentage score^43^.

Koel’s pyramid is used to classify the need for analytical procedures based on their utility and resource consumption^63^. The Need score of the proposed method is 100%, reflecting the method’s classification as simple, low solvent consumption, energy and resource efficiency, making it suitable for routine analysis with modest resource needs. On the contrary, the traditional chromatographic techniques are at the top of the pyramid with a 25% need score because these techniques necessitate specialized handling as well as extensive usage of chemicals and solvents. Whiteness, or “Quality,” is assessed using the RGB12 Algorithm. This approach looks for methods that are economical, environmentally friendly and offer great accuracy and precision. The Quality score is 96%, according to the method’s WAC profile in comparison to other reported chromatographic methods. In addition to a sustainable score of 76 due to the alignment with 13 of the SDGs, as detailed in Table S6.

The implementation of the proposed methodology using the NQS tool yielded the results summarized in Table 7. The overall score is 91%, demonstrating the voltammetric method’s durability, necessity and quality in the field, while highlighting its exceptional performance and crucial significance in accomplishing the SDGs.

**Table 7:** NQS index data output.

Need	100
Quality	96
Sustainability	76
NQS index	91

This study highlights the importance of aligning with the UN SDGs and demonstrates how scientific innovation may be effortlessly interwoven with environmental sustainability. It establishes a benchmark for integrating sustainability into scientific research. This study not only advances analytical chemistry but also serves as a strong call to action for the scientific community to strengthen its commitment to the 2030 global goals.

## Conclusion

A simple, rapid and Eco-friendly DP voltammetric method was successfully developed for the synchronized micro-estimation of PCT with ACL or DIC in fixed-dose combinations. The use of an unmodified GCE enabled sensitive and selective analysis without the need for electrode modification, organic solvents or complex sample preparation. The method demonstrated excellent validation performance in accordance with ICH guidelines and was effectively applied to pharmaceutical dosage forms. The principal novelty of this work lies in being the first voltammetric approach capable of simultaneously determining PCT in two clinically relevant binary mixtures using a single analytical platform, while integrating comprehensive multihued sustainability assessment tools alongside the NQS index. This dual focus on analytical performance and sustainability represents a significant advancement over conventional chromatographic techniques. Despite its advantages, the method is limited to electroactive compounds and aqueous buffer systems, which may restrict its applicability to certain formulations. Future research may focus on extending the approach to biological studies, other multicomponent pharmaceutical systems, exploring miniaturized or portable electrochemical platforms and integrating chemometric tools to further enhance selectivity and robustness. Overall, the proposed method offers a cost-effective, time-efficient and environmentally responsible alternative for routine quality control analysis, supporting both regulatory compliance and sustainable pharmaceutical practice.

## Supplementary Information

Below is the link to the electronic supplementary material.

Supplementary Material 1

## Data Availability

All data generated or analyzed in this study are fully included within this published article [and its supplementary information files].

## Abbreviations

AGREE: Analytical GREEnness
BAGI: Blue applicability grade index
BRB: Britton-Robinson buffer
COX: Cyclo-oxygenase
DP: Differential pulse
E_p_: Peak potential
GAC: Green analytical chemistry
GCE: Glassy carbon electrode
ICH: International Conference on Harmonization
I_p_: Peak current
LOD: Limit of detection
LOQ: Limit of quantitation
NQS: Need, quality and sustainability
RGB: Red…Green…Blue
SDGs: Sustainable development goals
UN: United Nations
WAC: White analytical chemistry
