# Supplementary material for: A multihued sustainable appraisal of the electrochemical method for synchronized micro-estimation of the household drug Paracetamol with Aceclofenac or Dicyclomine
Source: Sci Rep. 2026 Mar 22;16:9673. doi: 10.1038/s41598-026-41215-w (PMC13009362; doi:10.1038/s41598-026-41215-w)
Supplement: Supplementary file 1 — Supplementary Material 1 [file 41598_2026_41215_MOESM1_ESM.docx]

**
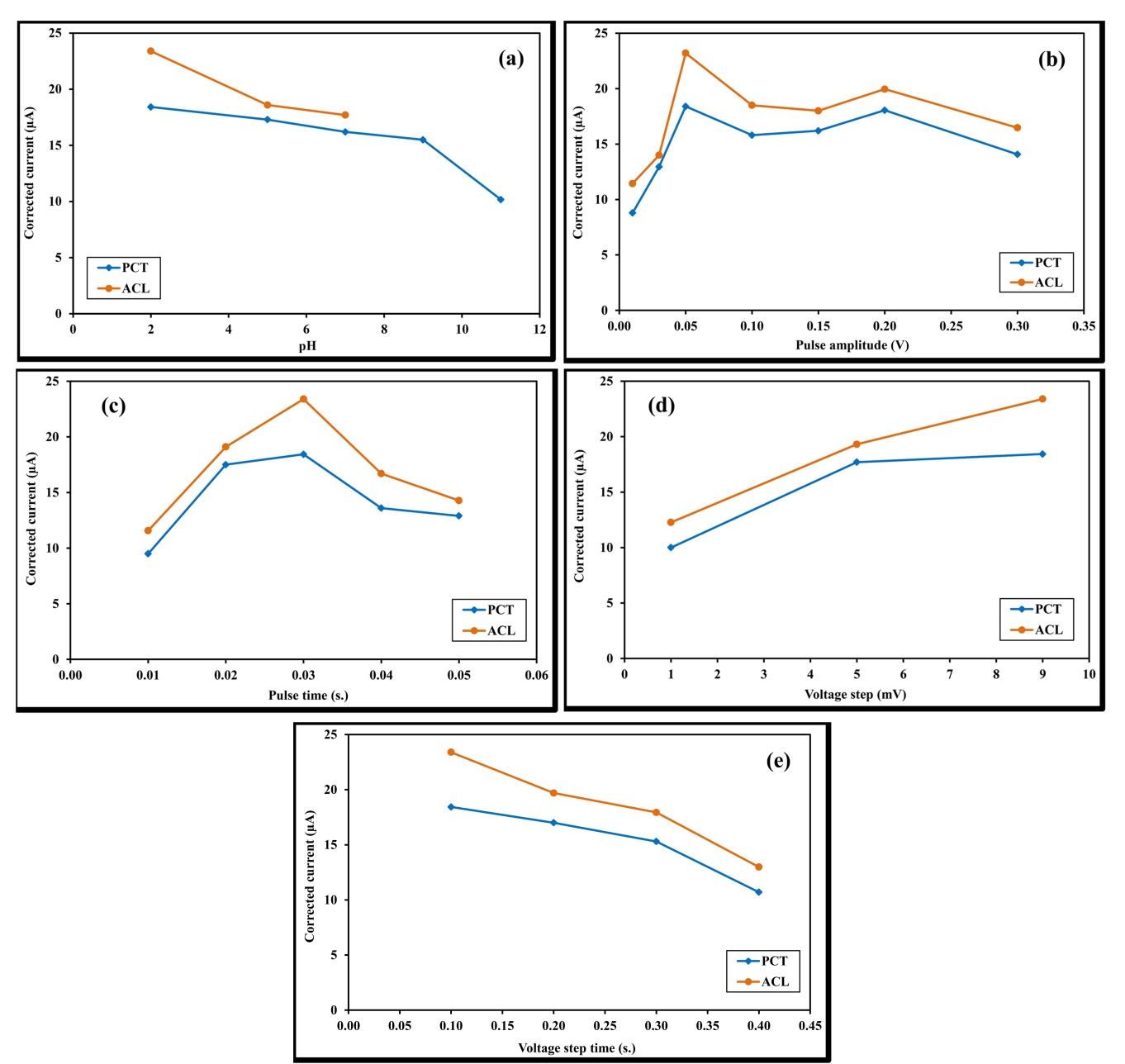
**

**Figure S1: Influence of (a) BRB pH (optimum at pH 2), (b) pulse amplitude (optimum is 0.05 V), (c) pulse time (optimum is 0.03 seconds), (d) voltage step (optimum is 9mV) and (e) voltage step time (optimum is 0.1 seconds) on the current response in differential pulse voltammetry for mixture 1 (10 µg. mL^-1^ of each drug) at a scan rate of** **100 mVs^-1^ versus Ag/AgCl reference electrode.**

**
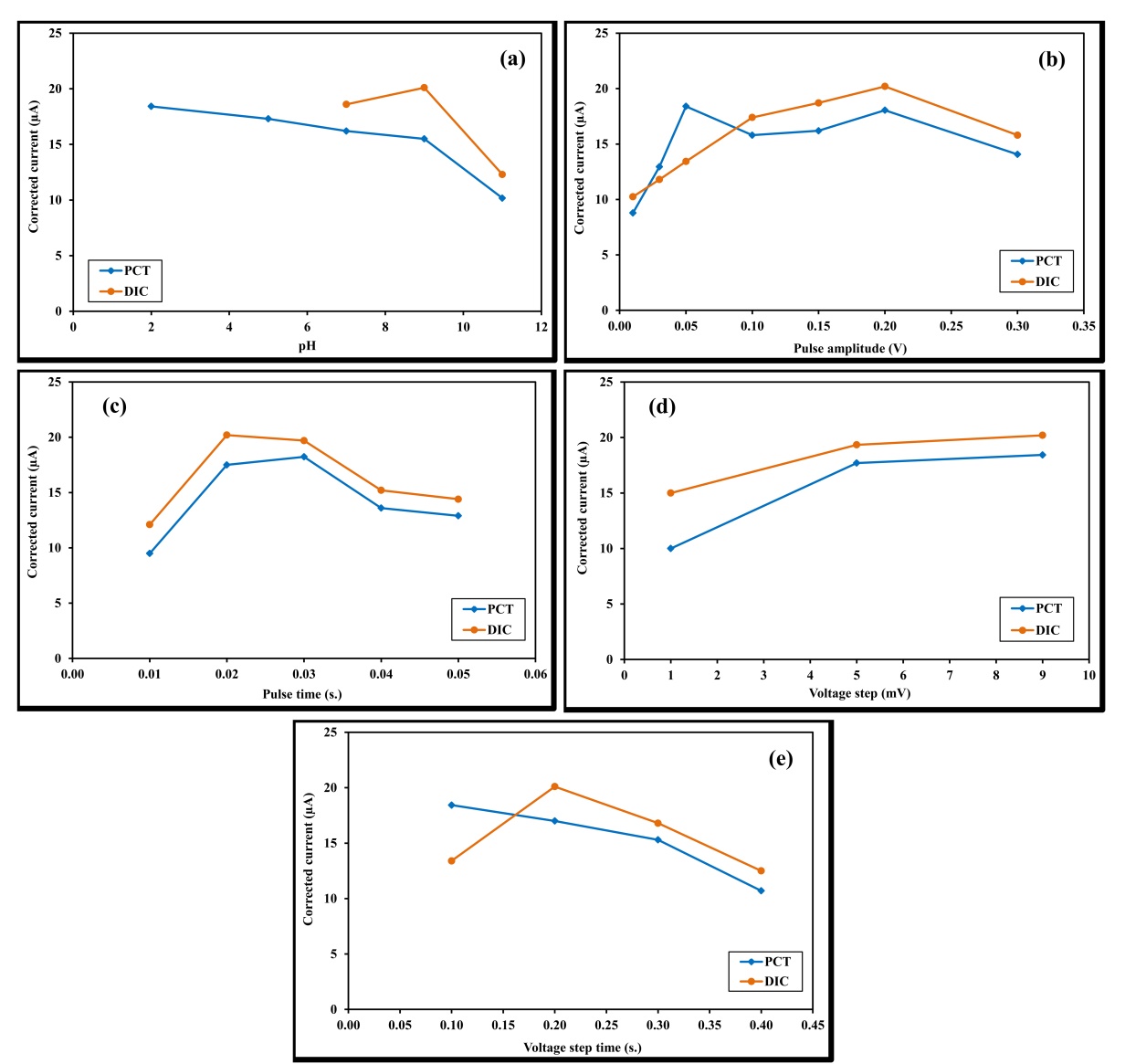
**

**Figure S2: Influence of (a) BRB pH (optimum at pH 9), (b) pulse amplitude (optimum is 0.2 V), (c) pulse time (optimum is 0.02 seconds), (d) voltage step (optimum is 9 mV) and (e) voltage step time (optimum is 0.2 seconds)on the current response in differential pulse voltammetry for mixture 2 (10 µg. mL^-1^ of each drug) at a scan rate of** **100 mVs^-1^ versus Ag/AgCl reference electrode.**

**(a)**

**(b)**

**(c)**

**Figure S3: Mechanism of electrochemical oxidation of (a) PCT, (b) ACL and (c) DIC.**

**(a)**

**
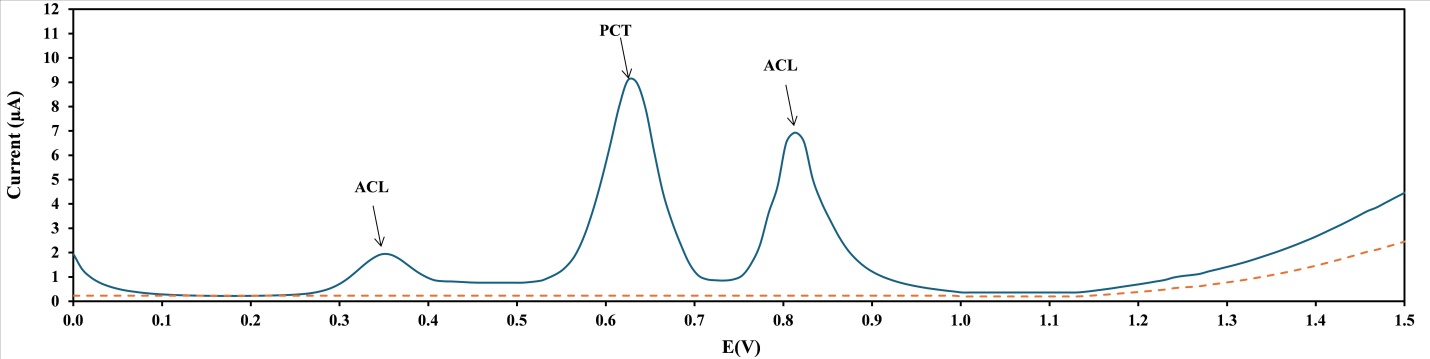
**

**(b)**

**
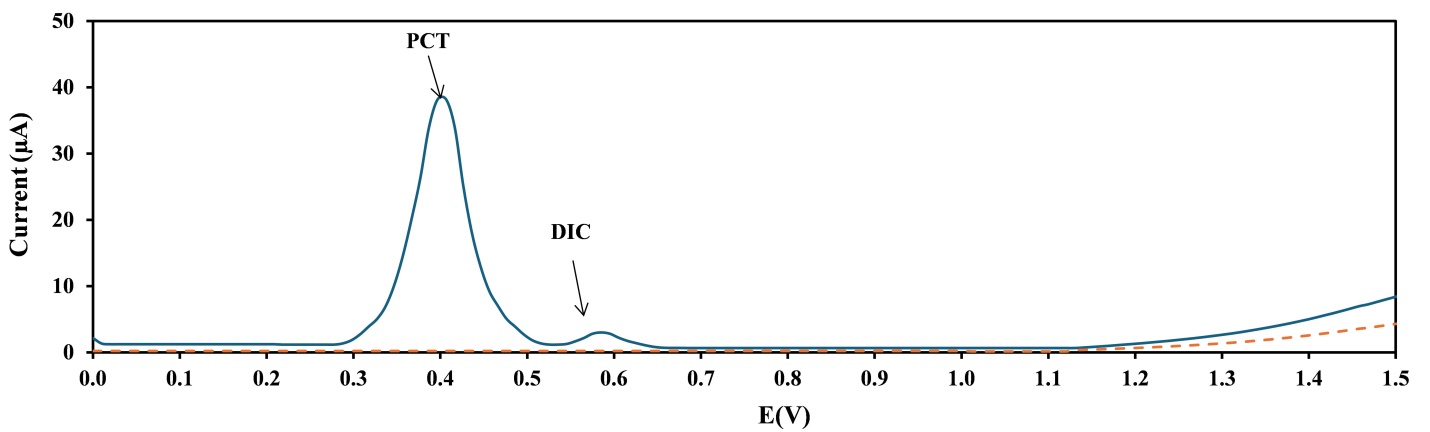
**

**Figure S4: Differential pulse voltammograms of laboratory-prepared tablet solutions of (a) mixture 1 (PCT-ACL) equivalent to 5:1 µg. mL^-1^using blank BRB (………) at pH 2, pulse amplitude of 0.05 V, pulse time of 0.03 seconds, voltage step of 9mV and voltage step time of 0.1 seconds, (b) mixture 2 (PCT-DIC) equivalent to 25:1 µg. mL^-1^ at pH 9, pulse amplitude of 0.2 V, pulse time of 0.02 seconds, voltage step of 9 mV and voltage step time of 0.2 seconds at a scan rate of** **100 mVs^-1^ versus Ag/AgCl reference electrode.**


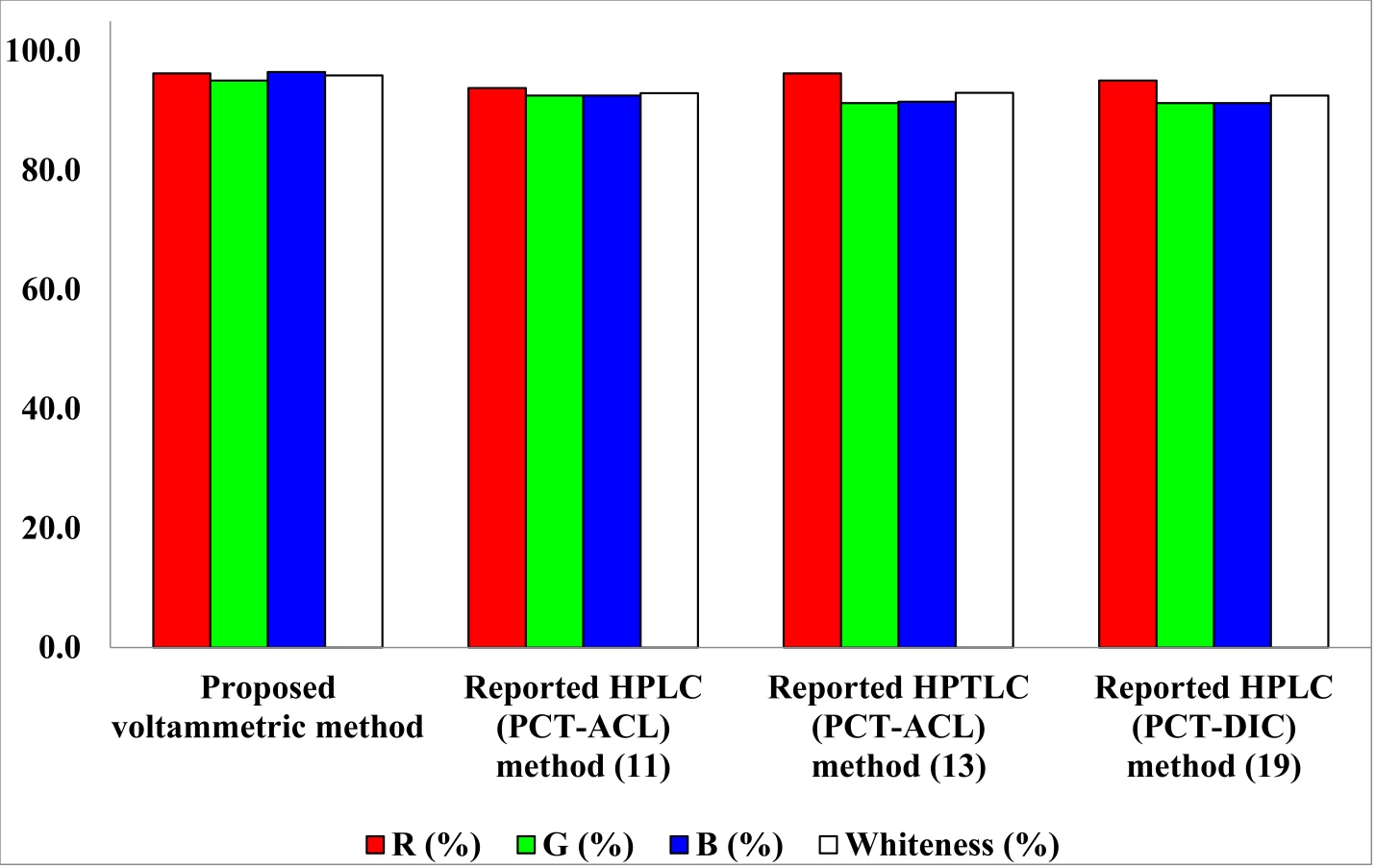


**Figure S5: RGB profiles for (a) the proposed voltammetric method, (b) reported HPLC(PCT-ACL)** [[11](#_ENREF_11)]**, (c) reported HPTLC(PCT-ACL)** [[13](#_ENREF_13)] **and (d) reported HPLC(PCT-DIC)** [[19](#_ENREF_19)] **methods.**

**Table S1: Robustness of the proposed voltammetric method with slight variation of the buffer pH**

| **Parameter** | **PCT** | | **ACL** | | **DIC** | |
| --- | --- | --- | --- | --- | --- | --- |
| **Buffer pH** | **E_p_** + **SD** | **RSD% of I_p_^a^** | **E_p_+SD** | **RSD% of I_p_ ^a^** | **E_p_+SD** | **RSD% of I_p_ ^a^** |
| **Mixture 1** | | | | | | |
| 2+0.2 | 0.62+0.008 | 0.03 | 0.81+0.007 | 0.04 |  | |
| **Mixture 2** | | | | | | |
| 9+0.2 | 0.41+0.008 | 0.06 |  | | 0.57+0.009 | 0.07 |

**^a^** Relative standard deviation percent of peak current of each drug for a concentration of 10 µg. mL^-1^ for PCT, ACL and DIC

**Table S2: Comparison of the proposed voltammetric method with other reported chromatographic methods** [[11](#_ENREF_11), [19](#_ENREF_19)]

|  | **Proposed method** | | **Reported method** | |
| --- | --- | --- | --- | --- |
| **Mixture 1** | **PCT** | **ACL** | **PCT** | **ACL** |
| **Mean % recovery ±SD*** | 99.92+0.28 | 99.67+0.19 | 100.03±0.13 | 99.80±0.10 |
| **%RSD** | 0.28 | 0.19 | 0.12 | 0.10 |
| **%Er** | -0.08 | -0.33 | 0.03 | -0.20 |
| **t- test** | 0.63 | 1.07 |  |  |
| **F- test** | 4.98 | 3.49 |  |  |
| **Mixture 2** | **PCT** | **DIC** | **PCT** | **DIC** |
| **Mean % recovery ±SD*** | 99.88+0.85 | 100.02+0.22 | 100.83±0.54 | 100.76±0.16 |
| **%RSD** | 0.85 | 0.22 | 0.54 | 0.16 |
| **%Er** | -0.12 | 0.02 | 0.83 | 0.76 |
| **t- test** | 1.74 | 5.08 |  |  |
| **F- test** | 2.45 | 1.94 |  |  |

^*^ Mean recovery of the found concentrations ± standard deviation for six determinations (n=6), t_tab_ =2.36, F_tab_ =19.30

**Table S3: Comparison of the proposed voltammetric method with other reported electrochemical and spectrophotometric methods**

| **Method** | **Linearity*** | **LOD*** | **LOQ*** | **r^2^** | **Reagent used** | **matrix** | **Remarks** | **Ref.** |
| --- | --- | --- | --- | --- | --- | --- | --- | --- |
| **Spectrophotometric**  **(PCT - ACL)** | PCT:3-15, ACL: 1-5 | PCT: 0.78, ACL: 0.28 | PCT: 2.35, ACL: 0.85 | 0.995, 0.994 | Methanol | Tablets | Application of a chemometric approach, which requires mathematical calculations | **[49]** |
| **Electrochemical method (PCT)** | 0.6-15 | 0.06 | 0.2 | 0.998 | Acetate buffer (4.51M) | Tablets | Determination of PCT only  No sustainability assessment | **[25]** |
| **Electrochemical method (PCT)** | 0.2-75.6 | 0.06 | **N/M** | 0.984 | Phosphate buffer (0.1M) | Tablets | Modified electrode with **multiple steps and materials** makes the method **more expensive**.  Determination of PCT only | **[29]** |
| **Electrochemical (ACL)** | 0.35-67.3 | 0.09 | **N/M** | 0.999 | Phosphate buffer (0.1M) | Tablets/ urine | Modified electrode with **multiple steps and materials** makes the method **more expensive**. | **[33]** |
| **Spectrophotometric method (PCT-DIC)** | PCT: 2-12, DIC: 10-35 | PCT: 0.09, DIC: 0.83 | PCT:0.29, DIC: 2.53 | 0.999, 0.998 | 0.1N NaOH | Tablets | Requiring mathematical calculations | **[23]** |
| **Electrochemical method (DIC)** | 0.92-6.18 | 0.19 | 0.59 | 0.995 | Britton Robinson buffer (0.04M) | Tablets, human urine and serum | Modified electrode with **multiple steps and materials** makes the method **more expensive**.  Detection of DIC only. | **[35]** |
| **Electrochemical method**  **(Mix 1 PCT- ACL, Mix2 PCT-DIC)** | **Mix 1**  0.2-25  **Mix 2**  1-25 | **Mix 1**  PCT:0.06  ACL:0.07  **Mix 2**  PCT:0.33  ACL:0.32 | **Mix 1**  PCT:0.18  ACL:0.2  **Mix 2**  PCT:0.99  DIC:0.97 | **Mix 1**  PCT:  0.9999  ACL:  0.9998  **Mix 2**  PCT:  0.9995  DIC:  0.9997 | Britton Robinson buffer (0.04M) | Tablets | Simultaneous determination of PCT with ACL or DIC | **Proposed method** |

***Concentrations in µg.mL^-1^**

**N/M: Not mentioned**

**Table S4: Analytical Eco-scale for assessment of greenness of the proposed voltammetric method compared with other reported chromatographic methods [**[**11**](#_ENREF_11)**,** [**13**](#_ENREF_13)**,** [**19**](#_ENREF_19)**]**

| **Reagent** | **Proposed voltammetric method** | **Reported**  **HPLC (PCT-ACL) method** [[11](#_ENREF_11)] | **Reported HPTLC (PCT-ACL) method** [[13](#_ENREF_13)] | **Reported HPLC (PCT-DIC) method** [[19](#_ENREF_19)] |
| --- | --- | --- | --- | --- |
| **Acetonitrile** |  | 8 |  | 8 |
| **Methanol** |  |  |  |  |
| **Acetic acid** | 4 |  | 4 |  |
| **Boric acid** | 2 |  |  |  |
| **Phosphoric acid** | 4 | 4 |  | 4 |
| **NaOH** | 2 |  |  |  |
| **Triethylamine** |  |  |  | 6 |
| **n-butanol** |  |  | 6 |  |
| **Ethyl acetate** |  |  | 8 |  |
| **Total penalty points for the reagents** | 12 | 12 | 18 | 18 |
| **Instrument** | | | | |
| **Energy** | 0 | 1 | 1 | 1 |
| **Waste** | 1 | 5 | 3 | 5 |
| **Occupational hazard** | 0 | 0 | 0 | 0 |
| **Final total penalty points (sum of reagent and instrument points)** | 13 | 18 | 22 | 24 |
| **Analytical Eco-scale score** | 87 | 82 | 78 | 76 |

**Table S5: Results of ChlorTox scores for the proposed method in comparison with reported methods** [[11](#_ENREF_11), [13](#_ENREF_13), [19](#_ENREF_19)] **in terms of the relative hazards with respect to chloroform (CH_sub_/CH_CHCI3_) derived using the WHN model**

| **Method** | **Stage** | **Compound** | **Relative hazard**  **(CHsub/CH_CHCl3_)**  **(WHN)** | **msub (g)** | **ChlorTox**  **(g)** | **Total ChlorTox**  **(WHN)** | **Ref­er­ence** |
| --- | --- | --- | --- | --- | --- | --- | --- |
| Proposed voltammetric method | Sample preparation and  voltammetric analysis | Phosphoric acid | 0.57 | 0.04 | 0.02 | 0.04 < 0.1 gram neglected | Pro­posed method |
|  |  | Acetic acid | 0.43 | 0.02 | 0.01 |  |  |
|  |  | Boric acid | 0.17 | 0.03 | 0.01 |  |  |
|  | | | | | | | |
| Reported HPLC  Method (PCT-ACL) | Sample preparation | Acetonitrile | 0.39 | 5.11 | 1.99 | 3.42 | [[11](#_ENREF_11)] |
|  | HPLC analysis | Acetonitrile | 0.39 | 3.65 | 1.43 |  |  |
|  |  | Phosphate buffer | 0 | 0.05 | 0 |  |  |
|  | | | | | | | |
| Reported HPTLC  Method (PCT-ACL) | HPTLC analysis | Ethyl acetate | 0.35 | 0.68 | 0.24 | 0.36 | [[13](#_ENREF_13)] |
|  |  | n-butanol | 0.61 | 0.20 | 0.12 |  |  |
|  |  | Glacial acetic acid | 0.43 | 0 | 0 |  |  |
|  | | | | | | | |
| Reported HPLC  Method (PCT-DIC) | Sample preparation | Acetonitrile | 0.39 | 5.90 | 2.30 | 5.26 | [[19](#_ENREF_19)] |
|  | HPLC analysis | Acetonitrile | 0.39 | 7.51 | 2.93 |  |  |
|  |  | Phosphate buffer | 0 | 0.04 | 0 |  |  |
|  |  | Triethylamine | 0.78 | 0.04 | 0.03 |  |  |

**Table S6: Impact of the sustainable voltammetric method on UN SDGs**

| **SDG** | **Application** |
| --- | --- |
| **Goal 3:** Good Health and Well-being | Designed for the multi-drug analysis that is essential for health monitoring, minimizing errors, and ensuring a better quality of life |
| **Goal 4:** Quality Education | Promotes scientific and educational developments, expanding the body of knowledge in sustainability and analytical chemistry, and creating new educational materials centered on sustainable methods devoid of organic solvents |
| **Goal 5:** Gender Equality | This project aims for gender balance in research collaborations |
| **Goal 7:** Affordable and Clean Energy | This technique is energy-efficient, affordable, and sustainable |
| **Goal 8:** Decent Work and Economic Growth | This method offers a practical approach to lower economic costs by using quicker and more effective analysis. |
| **Goal 9:** Industry, Innovation and Infrastructure | This method improves collaborations between academic research and the pharmaceutical industries by providing a versatile method for evaluating various dosage forms while also saving a great deal of time and money. |
| **Goal 10**: Reduced Inequality | Provides an affordable, effective, and easily accessible method of laboratory quality assurance that is particularly advantageous for labs in underdeveloped nations or those without access to pricey analytical equipment |
| **Goal 11**: Sustainable Cities and Communities | Enhances urban lab efficiency and lowers pollutants while promoting urban sustainability through the use of eco-friendly solvents and low energy. |
| **Goal 12**: Responsible Consumption and Production | Operates without the use of dangerous organic solvents, demonstrating resource sustainability and getting rid of harmful solvent use |
| **Goal 13**: Climate Action | Mitigates the environmental footprint of analytical procedures and energy consumption, which helps with climate action. |
| **Goal 14**: Life Below Water | Prevents toxic solvents and encourages environmentally friendly analytical methods to save aquatic life. |
| **Goal 15**: Life on Land | Helps preserve terrestrial ecosystems by using less energy and solvent. |
| **Goal 17**: Partnerships for the Goals | Analyzing various medication formulations with a single approach simplifies the process, saving time and money while promoting increased collaboration between pharmaceutical industries and academic institutions. |
